# Do empowerments influence experiencing intimate partner violence (IPV)? A multi-continental study of women across low and lower-middle-income countries

**DOI:** 10.1186/s12889-025-22027-5

**Published:** 2025-03-06

**Authors:** M Z E M Naser Uddin Ahmed, MD. Nahid Hassan Nishan, Helene Dahlqvist, Saidur Rahman Mashreky, Koustuv Dalal

**Affiliations:** 1 Department of Public Health, North South University, Dhaka, Bangladesh; 2 Division of Public Health Science, School of Health Sciences, Mid Sweden University, Sundsvall, Sweden

**Keywords:** Domestic violence, Empowering women, Economic empowerment, IPV, LMIC, Women’s empowerment

## Abstract

**Background:**

Distinct regional variations are observed in terms of factors influencing intimate partner violence (IPV) when women are empowered. This is a noticeable gap, and no comprehensive studies explore these influencing factors across different continents. Therefore, this study aimed to assess the relationship between women’s empowerment, autonomy, and IPV across different continental regions.

**Method:**

The Demographic and Health Survey (DHS) dataset across 26 countries with low and lower-middle-income countries was used in this study. We focused on a sample of 56,175 women aged 15–49 who had experienced IPV. For data analysis purposes, we have used the Chi-square test and binary logistic regression using Stata. We also account for complexities related to the survey and incorporate sampling weights.

**Results:**

Among 56,175 women from all six continents, 22,236 experienced IPV. IPV prevalence varies across regions, ranging from 12.81% in Cambodia [95% CI: 11.75%-13.92%] to 69.61% in Sierra Leone [95% CI: 66.93%-72.18%], with an overall prevalence of 39.46%. Empowered women demonstrated reduced odds of experiencing IPV, with notable protective effects in regions like Sub-Saharan Africa (e.g., Burundi: OR: 0.36, 95% CI: 0.29–0.44) and South and Southeast Asia (e.g., Cambodia: OR: 0.38, 95% CI: 0.30–0.50), However, exceptions such as Nigeria (OR: 1.53, 95% CI: 1.30–1.79) underscore regional disparities in the impact of empowerment.

**Conclusions:**

Empowering women socially through employment opportunities and autonomy significantly reduces the prevalence of IPV. Notably, women living below the poverty line and with limited education face heightened vulnerability. It is crucial for policymakers, organizations, and communities to utilize these findings to create more comprehensive environments for working women.

## Introduction

Intimate partner violence (IPV) is a global burden that poses a severe and urgent challenge to societal well-being. There are several complex factors that contribute to women’s increased susceptibility to physical and sexual abuse [1–3]. However, there are many concepts of women’s empowerment and its influencing likelihood of ever experiencing IPV in low and lower-middle-income countries. Women’s empowerment is an idea that involves different aspects, such as economics, and society. [4, 5]. A study in Togo highlights how structural and socio-economic conditions shape women’s risk of experiencing IPV, emphasizing the protective role of empowerment [6]. When women gain independence, access education, and have control over resources and decision-making, they are less vulnerable to violence [7, 8].

Economic empowerment is a critical aspect of women’s autonomy and independence. Research consistently demonstrates that women who have resources and independence are at a lower risk of encountering physical violence [9, 10]. For instance, a study conducted in India revealed that when women make contributions to their households, it decreases the likelihood of violence being inflicted upon them [11]. Another study has a similar finding: women may be protected against IPV through economic empowerment and better education [7]. However, research in Kenya revealed that IPV during pregnancy is influenced by socio-economic disparities, highlighting the intersection of economic vulnerability and life-stage-specific risks [12]. Social empowerment enhances women’s status, decision-making power, and access to education and healthcare [13].

In some instances, IPV is also normalized in women as male dominance is prevalent in some countries, and women accept IPV as a means of resolving disputes [14, 15]. However, the active participation of women in various decision-making processes within their families is closely connected with a significant reduction in the chance of encountering violent behavior [16]. While economic and social empowerment are crucial determinants, they operate within the broader context of deeply ingrained gender norms and attitudes [17–21]. Experts found that gender-inequitable attitudes among men are associated with higher levels of violence against women, even among economically empowered women. [16].

Economic independence through employment strengthens women’s self-esteem and bargaining power, reducing their risk of experiencing violence [22]. When women earn more than their partners, they may encounter pushback or resistance from their partners [23]. This resistance can manifest in various ways, including psychological abuse and physical violence, as men may feel their masculinity is threatened [24]. The level of independence women’s contribution in making decisions about their lives and bodies has an influence on how likely they are to experience IPV. [16, 25]. When women have autonomy, they can speak up for their rights, get help when needed, and make choices that protect their safety and well-being [25]. So, in terms of different country contexts, the influence of women empowerment in IPV is seen in different literature where there are distinct variations present based on the regions and the continent, but it is possible to make them one and find out the distinct pattern across all of them which creates a noticeable research gap, and there are no comprehensive studies that explore these influencing factors across different continental regions. Therefore, this study aims to uncover the relationship between women’s empowerment and IPV across different continental regions. The novelty of this research lies in its examination of the relationship between women’s empowerment and IPV across multiple continents, offering a comparative perspective rarely explored in previous studies. By integrating data from diverse socio-economic contexts, this study reveals global patterns and variations in how empowerment influences IPV, providing actionable insights for targeted interventions.

## Methodology

### Study description

For this study, we identified the secondary dataset library from the Demographic and Health Survey (DHS). DHS has gathered data from different regions which include 92 countries [26]. However, according to World Bank categorization, we specifically focused on 62 countries classified as low- and lower-middle-income. From these countries, we ultimately chose 26 countries that provided data on our area of interest – IPV. The list of countries that we included in our study are:

**Sub-Sahara African region**: Angola 2015–16 [27], Benin 2017–18 [28], Burundi 2016–17 [29], Cameroon 2018 [30], Comoros 2012 [31], Cote d’Ivoire 2021 [32], Ethiopia 2016 [33], Gambia 2019–20 [34], Liberia 2019–20 [35], Madagascar 2021 [36], Malawi 2015–16 [37], Mali 2018 [38], Nigeria 2018 [39], Sierra Leone 2019 [40], Tanzania 2015–16 [41], Togo 2013–14 [42], Uganda 2016 [43], Zambia 2018 [44], Zimbabwe 2015 [45].

**South and Southeast Asian region**: Afghanistan 2015 [46], Cambodia 2021–22 [47], Myanmar 2015–16 [48], Nepal 2022 [49]

**Central Asian region**: Kyrgyz Republic 2012 [50], **Latin America and Caribbean region**: Haiti 2016–17 [51], Honduras 2011–12 [52]

### Study design

For this study, we used cross-sectional data from the DHS. Since we used a secondary dataset from the DHS survey, our specific focus was on variables related to women’s empowerment and IPV which were found on the DHS dataset in countries with low incomes and lower-middle-range economies. The surveys conducted by the DHS follow a two-step sampling approach that relies on enumeration areas (EAs) to gather information. The data collection process adheres to the guidelines established by the EA framework and includes aspects such as sample size, sample selection, survey budget, schedules, field staff recruitment, training, supervision, logistics, and ethical clearance. The DHS program conducts regional workshops to train national implementing agencies on the DHS methodology, and the national agencies then conduct in-country training for field staff, who are responsible for conducting the interviews [53].

### Study participant

We used individual recorded information (IR files) sourced from the DHS, which contains data from women between the ages of 15 and 49. Our focus was determining if women had encountered any IPV in their lives. To get accurate results, we first selected the appropriate variables, then adjusted them correctly, removed any missing values, and eliminated any irrelevant variables. In this study, we had a total of 56,175 participants (weighted) from all continents around the world. To conduct our analysis, we ensured that the sample was representative by applying appropriate weights to each region’s samples and adjusted according to population sizes and survey years for each country.

### Outcome variable

The outcome variable focuses on the variables that determine women’s ever experiences of physical or sexual violence. Physical violence refers to any form of force that causes a woman pain, injury, or discomfort, such as slapping, pushing, hitting, kicking, choking, or burning. Sexual violence encompasses acts of unwanted sexual activity, including being forced into sex or engaging in acts. These factors are derived from the DHS, which provides information on instances of violence experienced by women from their partners and others outside those relationships. The data categorizes these experiences as variables indicating whether a woman has encountered physical violence, sexual violence, or both at any point in her conjugal life. Every form of violence is finally merged and labeled as a binary variable. A value of “1” represents experiencing violence, while a value of “0” indicates not experienced.

### Independent variable

In this study, all the independent variables were categorical, and most of them were categorized by DHS itself. For demographics, we used these variables: age group, resident, wealth index, educational level, have insurance, and head of the household. These variables are categorized in the following way: age is categorized to 15–19 y, 20–24 y, 25–29 y, 30–34 y, 35–39 y, 40–44 y, 45–49 y. The resident variable is categorized into Rural and Urban. Furthermore, the wealth index contains five categories: Poorest, Poorer, Middle, Richer, and Richest. Education level is categorized as No education, Primary, Secondary, and Higher. Having insurance variable was categorized as binary: Yes, or No. Also, the Head of the household variable was divided into Male and female. The empowerment variables contain five variables. The working status was recoded to a binary measure and labeled as working and not working. The employment status variable was categorized into three categories: seasonal, all-year, and occasional. Another variable, the respondent earning gap, was categorized as Partner no income, More than her, Less than her, Same as her. The variable of respondent autonomy gauges the level of decision-making authority that a woman possesses in aspects of her life. Six other variables from the DHS dataset were combined to construct this variable. These variables inquire about who makes decisions regarding the woman’s healthcare, significant household purchases, daily household needs, visits to family or relatives, daily food choices, and allocation of her partner’s earnings. The respondent autonomy variable is divided into four categories: Respondent alone, Respondent and partner together, Partner alone, and Someone else. These categories represent combinations of responses provided by women for the six decision-making variables. The objective behind creating this variable is to capture women’s autonomy within their households by examining each decision separately. Finally, the respondent spending decision variable was also included in our analysis, and this is categorized as Respondent alone, Respondent and partner, Partner alone, and Someone else.

### Data analysis

Descriptive analysis was conducted to summarize the demographic characteristics of the participants. The Chi-square (χ^2^) test was performed to identify any associations between dependent and independent variables. Subsequently, binary logistic regression was used to determine significant associations between IPV and other determinants. A 95% confidence interval was maintained, and the adjusted odds ratios (AOR) with significance levels were presented. Significance is indicated with asterisks (*), where more asterisks denote stronger significance levels. The statistical software used for the analysis was Stata version 17. The complex survey design of the DHS dataset was considered by incorporating sampling weights, stratification, and clustering into the analysis. Weighting ensures reliable and valid estimates by adjusting for potential biases, such as non-response. This approach, recommended by the Guide to DHS Statistics, provides robust results that account for the survey design [54].

To test the direct influence of women’s empowerment on IPV, a binary variable for women’s empowerment (empower) was created. Empowerment was assessed based on two dimensions: decision-making participation and rejection of wife-beating. Women were classified as “empowered” if they participated in decisions regarding their own health care, large household purchases, and visits to family or relatives, either alone or jointly with their husbands, and rejected all justifications for wife-beating. Rejection of justification for IPV was assessed by examining whether women rejected all specified scenarios where wife-beating might be considered acceptable, such as burning food, arguing with their husband, going out without informing him, neglecting children, or refusing sexual relations. Women not meeting both criteria were classified as “not empowered,” while cases with missing data were excluded. This approach aligns with the DHS framework for measuring women’s empowerment [55].

The AOR, confidence intervals (CI), and *p*-values for each covariate were displayed and interpreted in the context of the research question. Visual representations, including tables and graphs, were used to summarize and present the findings effectively.

## Result

### Demographic characteristics of the participants

In our study, exposure to IPV among women in various countries on multiple continents is displayed in different demographic characteristics such as age group, residence, wealth index, etc. In Sub-Saharan Africa, IPV varies by age group, with the highest among women aged 30–34 (up to 24.96%). Rural areas have higher percentages compared to urban areas, and there are variations across wealth categories and education levels. Details information is provided in (Table 1).

**Table 1:** Demographic characteristics of the participants

Sub-Sahara Africa																			
Characteristics	**Angola**	**Benin**	**Burundi**	**Cameroon**	**Comoros**	**Cote d’Ivoire**	**Ethiopia**	**Gambia**	**Liberia**	**Madagascar**	**Malawi**	**Mali**	**Nigeria**	**Sierra Leone**	**Tanzania**	**Togo**	**Uganda**	**Zambia**	**Zimbabwe**
**Age Group**																			
15–19 y	4.60	3.51	1.92	3.41	3.12	2.67	5.15	3.43	3.05	6.48	5.64	7.39	3.15	4.50	4.06	1.52	5.83	3.77	3.28
20–24 y	14.22	13.08	13.73	14.72	11.11	12.73	12.70	10.44	11.18	16.92	18.15	15.99	10.53	12.78	15.46	12.00	20.33	13.04	12.88
25–29 y	21.55	23.83	23.20	22.25	19.78	16.74	26.31	20.76	16.16	18.93	21.26	21.28	20.62	21.23	19.96	22.65	20.67	18.43	19.90
30–34 y	16.75	19.94	22.69	22.29	20.31	24.52	22.54	21.49	21.36	17.61	21.00	19.00	20.93	17.11	18.34	21.01	18.24	20.47	24.96
35–39 y	17.24	16.04	17.34	16.94	24.45	19.76	15.15	18.22	21.30	15.13	16.57	17.69	21.30	21.49	18.50	18.85	15.63	20.51	17.97
40–44 y	15.88	13.39	12.75	10.62	12.40	14.33	9.87	14.39	12.88	14.34	11.21	11.24	12.32	13.59	14.14	13.51	10.46	13.91	12.84
45–49 y	9.76	10.21	8.38	9.78	8.82	9.25	8.28	11.28	14.07	10.59	6.16	7.41	11.16	9.31	9.53	10.47	8.85	9.88	8.18
**Resident**																			
Rural	33.37	59.19	86.89	50.54	69.60	46.22	63.80	28.29	40.09	80.11	74.04	71.98	50.68	56.22	58.26	54.70	76.53	53.85	55.80
Urban	66.63	40.81	13.11	49.46	30.40	53.78	36.20	71.71	59.91	19.89	25.96	28.02	49.32	43.78	41.74	45.30	23.47	46.15	44.20
**Wealth index**																			
Middle	19.34	21.33	18.25	19.43	18.44	19.43	12.94	22.02	19.41	20.70	18.15	20.27	21.15	19.68	16.70	18.37	21.03	18.87	16.73
Poorest	17.37	14.36	21.27	18.85	14.73	21.59	12.56	18.88	18.07	16.44	15.22	12.97	14.99	18.87	11.88	16.76	15.99	15.51	11.99
Poorer	18.67	18.03	19.95	20.44	18.48	18.61	14.67	18.07	17.65	17.41	16.25	19.66	17.30	18.38	14.27	16.42	18.96	17.97	15.10
Richer	18.95	23.18	17.63	19.81	24.91	20.32	18.14	21.84	21.46	21.90	17.78	24.47	22.01	23.85	23.65	23.25	20.42	22.24	26.96
Richest	25.66	23.11	22.91	21.48	23.44	20.05	41.69	19.19	23.42	23.56	32.61	22.63	24.55	19.22	33.50	25.19	23.60	25.42	29.22
**Education level**																			
No education	24.59	65.82	49.47	21.46	39.01	61.10	42.39	44.27	41.36	16.55	11.53	67.59	34.74	57.46	12.74	38.30	11.48	7.56	1.26
Primary	41.10	18.78	35.25	35.46	21.54	20.90	29.72	14.80	20.51	47.43	54.09	14.64	17.58	13.55	65.07	36.52	56.90	48.14	24.81
Secondary	28.01	14.40	14.01	36.56	25.77	14.87	10.90	32.66	33.71	32.49	25.80	15.41	35.24	23.60	19.87	23.45	21.46	35.06	61.73
Higher	6.30	0.99	1.28	6.53	13.67	3.13	17.00	8.27	4.42	3.53	8.58	2.36	12.43	5.39	2.32	1.73	10.16	9.25	12.20
**Have insurance**																			
No	96.56	98.73	68.66	96.94	93.61	90.55	94.53	96.45	93.62	95.53	96.76	92.54	96.75	92.90	86.84	93.36	97.80	95.67	82.32
Yes	3.44	1.27	31.34	3.06	6.39	9.45	5.47	3.55	6.38	4.47	3.24	7.46	3.25	7.10	13.16	6.64	2.20	4.33	17.68
**Head household**																			
Male	81.26	83.34	85.72	84.41	63.69	86.79	79.69	81.83	78.21	92.89	82.46	87.85	88.78	77.94	88.26	82.51	81.06	90.50	69.04
Female	18.74	16.66	14.28	15.59	36.31	13.21	20.31	18.17	21.79	7.11	17.54	12.15	11.22	22.06	11.74	17.49	18.94	9.50	30.96
**Working status**																			
Not Working	1.90	1.84	3.95	4.69	12.92	6.91	17.48	11.05	4.81	3.63	7.51	3.72	4.56	3.44	4.74	2.25	5.09	10.25	19.01
Working	98.10	98.16	96.05	95.31	87.08	93.09	82.52	88.95	95.19	96.37	92.49	96.28	95.44	96.56	95.26	97.75	94.91	89.75	80.99
**Employment status**																			
Seasonal	91.19	71.69	73.12	61.56	65.83	73.42	60.83	62.39	82.64	75.29	48.19	52.79	83.14	85.97	55.61	78.42	63.59	55.82	56.48
All year	4.22	19.50	11.77	26.91	15.91	17.54	22.63	33.41	13.98	19.00	43.54	36.33	12.83	11.29	38.77	12.82	30.86	38.12	27.71
Occasional	4.59	8.81	15.11	11.53	18.26	9.03	16.54	4.20	3.38	5.71	8.28	10.88	4.03	2.74	5.62	8.76	5.55	6.06	15.81
**Respondent earning gap**																			
Partner no income	11.27	7.72	9.04	4.78	31.18	5.92	18.25	8.95	11.02	10.16	8.24	5.76	4.94	9.88	9.69	8.23	9.95	9.70	12.63
More than	72.75	83.32	74.36	83.56	58.52	88.25	59.14	84.32	69.84	51.39	73.76	89.35	85.60	77.31	67.97	84.73	76.35	70.76	68.11
Less than	12.41	8.16	15.32	10.23	8.34	4.85	19.41	3.71	14.59	37.13	12.11	2.79	8.89	11.04	20.53	6.52	12.69	16.24	16.17
Same	3.56	0.81	1.28	1.43	1.96	0.98	3.21	3.03	4.55	1.32	5.89	2.10	0.57	1.76	1.82	0.52	1.01	3.30	3.10
**Respondent autonomy**																			
Respondent alone	4.48	1.96	2.73	1.71	10.42	1.73	7.86	2.11	6.83	2.77	2.91	1.87	1.11	2.47	1.34	0.91	3.27	4.06	3.88
Respondent and partner	41.70	17.10	50.62	39.01	21.27	17.11	59.46	8.50	62.89	70.45	38.83	4.24	23.55	31.02	36.88	10.95	34.71	51.32	61.25
Partner alone	52.12	80.16	46.41	58.76	59.29	80.53	31.87	85.71	28.98	26.15	57.06	90.94	75.10	65.65	60.64	87.23	61.37	44.17	33.56
Someone else	1.70	0.78	0.23	0.51	9.03	0.63	0.81	3.68	1.29	0.63	1.21	2.94	0.24	0.86	1.14	0.91	0.65	0.45	1.31
**Spending decision**																			
Respondent alone	39.78	73.02	21.90	52.89	36.15	67.30	31.53	81.29	20.84	30.18	28.23	79.97	70.96	36.81	36.01	89.15	52.54	29.43	31.99
Respondent and Partner	41.22	19.04	65.28	38.95	25.52	18.40	55.90	9.70	67.67	65.21	45.59	6.16	18.81	34.69	55.44	7.10	39.09	51.21	62.60
Partner alone	17.76	7.78	12.74	8.09	37.22	14.23	9.56	8.58	10.95	4.50	26.10	13.11	8.23	28.43	7.54	3.71	7.97	19.18	5.19
Someone else	1.24	0.17	0.08	0.06	1.11	0.06	3.01	0.42	0.55	0.11	0.08	0.76	2.00	0.08	1.00	0.04	0.41	0.18	0.21
Total	2482.00	3573.00	2399.00	2020.00	626.00	2590.00	839.00	902.00	783.00	4017.00	1105.00	1285.00	4633.00	1178.00	2748.00	2781.00	3439.00	2528.00	2287.00

**Fig. 1:**
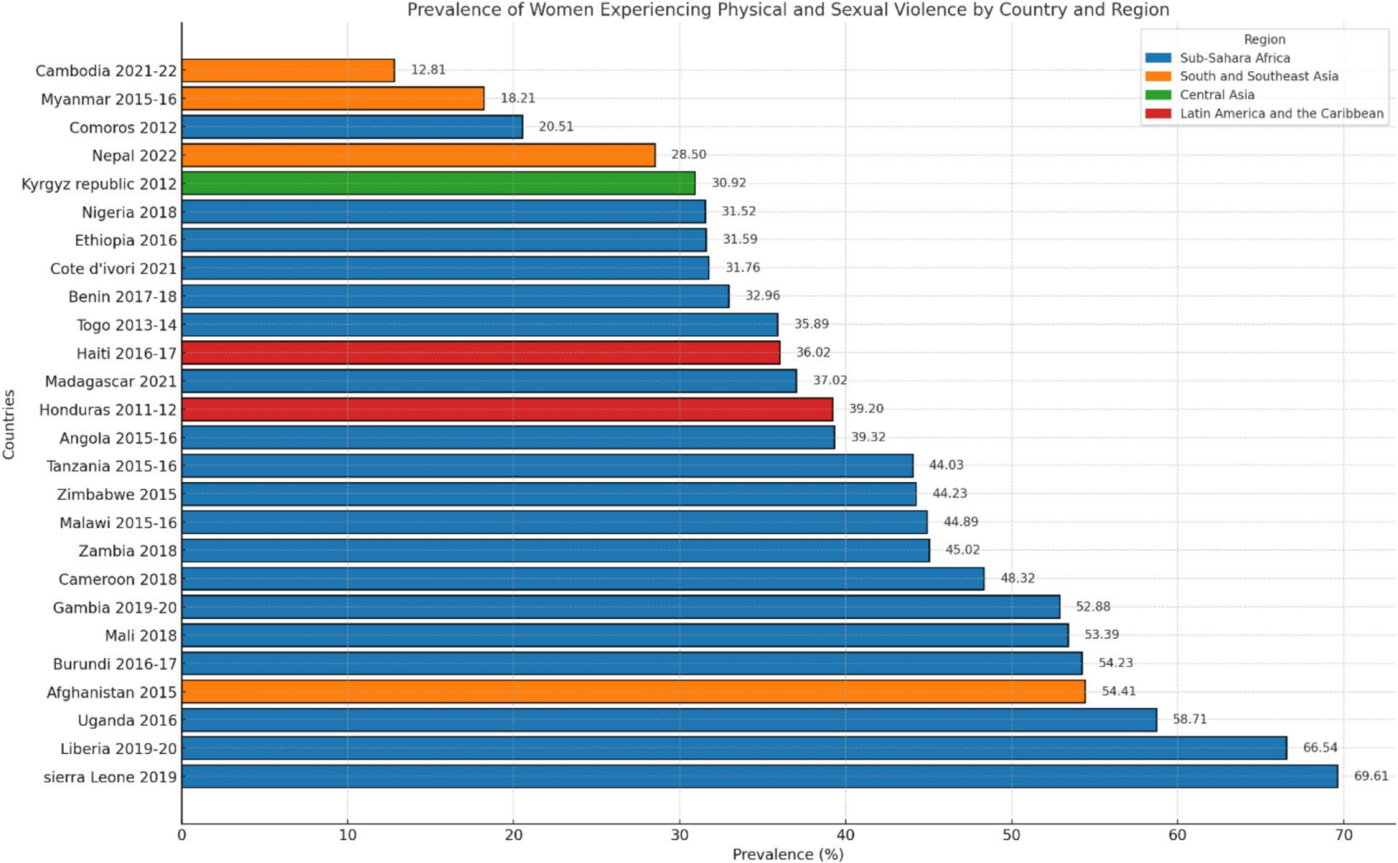
Horizontal bar chart showing the prevalence (%) of women experiencing physical and sexual violence, with regional and country-specific variations labeled

In Sub-Saharan Africa, the prevalence of IPV varies, with Sierra Leone showing the highest at 69.61% [95% CI: 66.93%−72.18%] and Comoros the lowest at 20.51% [95% CI: 17.36%−23.68%]. Liberia and Mali stand out at 66.54% [95% CI: 62.89%−69.52%] and 53.39% [95% CI: 50.63%−56.09%], respectively. In South and Southeast Asia, Afghanistan shows the highest at 54.41% [95% CI: 51.90%−56.84%], while Cambodia has the lowest at 12.81% [95% CI: 11.75%−13.92%]. The Latin America and Caribbean regions show a prevalence ranging from 36.02% [95% CI: 34.15%−37.98%] in Haiti to 39.20% [95% CI: 37.68%−40.65%] in Honduras. The Kyrgyz Republic in Central Asia shows a prevalence of 30.92% [95% CI: 28.28%−33.76%]. Overall, an alarming average of 39.46% of women across all regions have experienced IPV (Fig. 1).

### Socio-demographic influencing IPV

Age plays a significant role in determining the likelihood of experiencing IPV. In Uganda, women aged 40–44 and 45–49 face notably higher odds of IPV, with AORs of 1.96 and 2.17 (CI: 1.29–2.99 and 1.31–3.60), respectively, compared to the reference group aged 15–19. Also, Zambia and Zimbabwe exhibit similar trends, with women aged 40–44 having elevated odds, with AORs of 2.32 CI: 1.30–4.16. The Kyrgyz Republic stands out significantly, with women aged 45–49 having a striking 12.69 times higher odds of experiencing IPV. Conversely, in Gambia, women aged 35–39 have significantly lower odds, with an AOR of 0.32, CI: 0.11–0.92. In Haiti, there is an observable pattern where women, between the ages of 35–39 and 40–44 have a likelihood of experiencing certain outcomes with odds ratios of 0.46 CI: 0.21–0.99 and 0.33 CI: 0.15–0.73 respectively. It is interesting to note that the type of residence also reveals intriguing patterns when it comes to violence. In Cote d’Ivoire individuals living in urban areas are found to have around 0.7 times the odds of experiencing violence compared to those residing in rural regions. In contrast, Malawi and Tanzania reveal higher odds for urban residents, with AOR of 1.91 and 1.38, CI: 1.13–3.25 and 1.01–1.88, respectively. Haiti and Honduras also show significant associations, with urban residents having 1.51 and 1.5 times the odds of experiencing IPV compared to those in rural areas.

The relationship between the wealth index and IPV varies across different countries. In Uganda, Zambia, and Myanmar, individuals in the richest wealth index category consistently have significantly lower odds of experiencing IPV, with AOR of 0.59 (CI: 0.43–0.83), 0.62 (CI: 0.40–0.95), and 0.47 CI: (0.26–0.84), respectively. On the contrary, Madagascar, Sierra Leone, and Honduras exhibit contrasting trends, with individuals in the poorest wealth index category displaying significantly lower odds of experiencing IPV compared to those in the middle wealth index category, with AORs of 0.7, 0.61, and 0.72, (CI: CI: 0.50–0.98, 0.38–1.00, 0.55–0.94) respectively. Togo shows a significantly higher odds ratio (AOR of 2.02 CI: 1.42–2.88) for the poorest wealth index category, while Uganda and Zambia also show higher AOR (1.48 CI: 1.14–1.91 & 1.61 CI: 1.15–2.24) for the poorest wealth index category.

Education levels also play a complex role in IPV. In countries such, as Angola, Cameroon, Madagascar, and Nigeria, individuals who have received secondary education are more likely to face violence compared to those without any formal education. For example, in the case of Cameroon having completed primary education (with an AOR 2.53 CI: 1.77–3.62) or secondary education (with AOR 3.42 CI: 2.21–5.32) is linked to increased chances of experiencing IPV. Conversely, in countries like Cambodia, Nepal, Haiti, and Honduras, individuals with primary and secondary education consistently display lower odds of experiencing IPV. In Cambodia, we can see that when it comes to violence having completed primary education (AOR of 0.64 CI: 0.45–0.92) or secondary education (AOR of 0.55 CI: 0.35–0.84) is linked to a lower likelihood. In Honduras, the situation is a bit more complex. While secondary education (AOR of 0.64 CI: 0.43–0.98) and higher education (AOR of 0.53 CI: 0.31–0.89) are associated with reduced odds of violence than those who don’t appear to have no education.

### Economic and employment factors influencing IPV

Examining various factors, such as the gender of the household head, working status, employment type, earning disparity, and spending decisions, provides insights into the complex relationship with IPV. In Angola, the gender of the household head significantly impacts the likelihood of IPV. Households led by females are 0.61 times less likely to experience IPV than those with male heads. Across countries like Cameroon, Togo, and Afghanistan, a consistent trend emerges—working women consistently demonstrate significantly lower odds of experiencing IPV compared to their non-working counterparts. For example, in Cameroon, the AOR for working women is 0.42 CI: 0.24–0.75, indicating a substantial reduction in the risk of IPV. Similar trends are observed in Togo (AOR: 0.51 CI: 0.30–0.86) and Afghanistan (AOR: 0.24 CI: 0.10–0.58), emphasizing the empowerment that economic independence can provide. However, in Madagascar and Zambia, individuals with occasional employment consistently exhibit significantly higher odds of experiencing IPV compared to those with seasonal employment, with AOR of 1.88 and 1.63 (CI: 1.21–2.91 and 1.06–2.52), respectively. In contrast, Nigeria and Honduras with AOR of 0.58 (CI: 0.46–0.73) and 0.75 (CI: 0.60–0.93) demonstrate a protective effect associated with stable year-round employment. Moving on the earning disparity between partners, as seen in Tanzania and Afghanistan, reveals intriguing patterns. In Tanzania, women who earn less than their partners exhibit significantly lower odds (AOR of 0.18 CI: 0.04–0.75) of experiencing IPV. A similar trend is observed for women earning the same amount as their partners. In Afghanistan, women who earn the same amount as their partners also experience significantly lower odds (AOR of 0.34 CI: 0.12–0.93) of IPV.

### Women’s autonomy and its role in IPV

Women’s autonomy, including decision-making and spending decisions, showed varied associations with IPV across countries. Decision-making autonomy significantly influenced IPV outcomes. For instance, in Burundi, women whose partners made decisions alone faced higher odds of IPV (AOR: 2.23, CI: 1.22–4.07), while in Nigeria and Zambia, partner-led decision-making was associated with lower odds (e.g., Nigeria: AOR: 0.58, CI: 0.46–0.73). Similarly, financial autonomy had nuanced effects. In Tanzania and Afghanistan, women earning less than or equal to their partners had reduced odds of IPV (Tanzania: AOR: 0.18, CI: 0.04–0.75; Afghanistan: AOR: 0.34, CI: 0.12–0.93). Spending decisions also revealed a complex pattern: in the Kyrgyz Republic and Nigeria, collaborative spending was linked to increased IPV risk, whereas in Honduras, it reduced IPV odds (AOR: 0.81, CI: 0.68–0.97). These findings highlight the context-specific nature of women’s autonomy and its influence on IPV (Table 2).

**Table 2:** Association and influence of factors towards IPV

Sub-Sahara Africa												
**Characteristics**	Angola			Benin			Burundi			Cameroon		
	Percent. %	AOR	95% CI	Percent. %	AOR	95% CI	Percent. %	AOR	95% CI	Percent. %	AOR	95% CI
**Age Group**	X^2^ = 4.49			X^2^ = 11.88			X^2^ = 14.82			X^2^ = 24.37^Ψ^		
**15–19 y**	4.02	Ref		3.29	Ref		1.82	Ref		3.27	Ref	
**20–24 y**	15.32	1.4	0.80–2.43	12.10	0.96	0.48–1.91	14.08	1.05	0.50–2.19	12.18	0.71	0.41–1.23
**25–29 y**	20.10	1.25	0.75–2.08	26.75	1.27	0.65–2.48	22.23	0.94	0.46–1.92	22.88	1.14	0.66–1.96
**30–34 y**	17.44	1.43	0.84–2.43	17.60	0.89	0.45–1.74	20.78	0.96	0.46–1.99	22.96	1.26	0.73–2.16
**35–39 y**	18.23	1.59	0.94–2.67	15.47	0.93	0.47–1.83	18.95	1.48	0.72–3.06	16.29	1.00	0.57–1.75
**40–44 y**	15.69	1.39	0.75–2.57	14.60	1.16	0.58–2.33	13.81	1.34	0.62–2.93	13.20	1.87	1.02–3.42*
**45–49 y**	9.19	1.34	0.71–2.53	10.18	0.95	0.46–1.96	8.34	0.95	0.43–2.09	9.22	1.05	0.58–1.89
**Resident**	X^2^ = 0.00			X^2^ = 3.34			X^2^ = 35.29^ΨΨΨ^			X^2^ = 2.89		
**Rural**	33.39	Ref		61.60	Ref		90.40	Ref		48.68	Ref	
**Urban**	66.61	1.13	0.75–1.69	38.40	0.89	0.68–1.15	9.60	0.99	0.68–1.46	51.32	0.84	0.65–1.10
**Wealth Index**	X^2^ = 8.28			X^2^ = 1.53			X^2^ = 96.48^ΨΨΨ^			X^2^ = 40.60^ΨΨ^		
**Middle**	21.88	Ref		21.14	Ref		18.30	Ref		20.87	Ref	
**Poorest**	18.73	1.26	0.77–2.06	14.28	0.94	0.64–1.37	24.73	1.30	0.98–1.73	13.55	0.85	0.56–1.27
**Poorer**	17.37	0.93	0.62–1.42	18.92	1.03	0.74–1.42	22.03	1.25	0.92–1.69	22.71	1.27	0.89–1.81
**Richer**	18.22	0.69	0.46–1.02	23.66	1.09	0.80–1.49	18.94	1.17	0.86–1.60	19.94	0.78	0.56–1.10
**Richest**	23.81	0.63	0.36–1.09	22.00	1.19	0.82–1.75	16.00	0.65	0.42–1.00	22.93	0.91	0.59–1.39
**Education level**	X^2^ = 12.91			X^2^ = 10.01^Ψ^			X^2^ = 0.53^ΨΨΨ^			X^2^ = 88.66^ΨΨΨ^		
**No education**	22.18	Ref		69.29	Ref		54.76	Ref		13.40	Ref	
**Primary**	41.98	1.34	0.94–1.90	17.87	0.84	0.64–1.10	35.91	0.89	0.72–1.10	38.50	2.53	1.77–3.62***
**Secondary**	31.23	1.97	1.21–3.21**	12.27	0.69	0.50–0.96*	8.99	0.57	0.37–0.87**	42.26	3.42	2.21–5.32***
**Higher**	4.61	1.37	0.60–3.12	0.58	0.40	0.15–1.07	0.33	0.22	0.06–0.83*	5.85	2.29	1.17–4.46*
**Have Insurance**	X^2^ = 10.31^Ψ^			X^2^ = 3.16			X^2^ = 10.99^Ψ^			X^2^ = 0.58		
**No**	98.25	Ref		99.26	Ref		71.35	Ref		97.23	Ref	
**Yes**	1.75	0.42	0.17–1.03	0.74	0.60	0.23–1.56	28.65	1.16	0.91–1.47	2.77	0.77	0.35–1.69
**Head Household**	X^2^ = 9.31^Ψ^			X^2^ = 0.17			X^2^ = 0.14			X^2^ = 0.00		
**Male**	84.70	Ref		82.93	Ref		85.49	Ref		84.46	Ref	
**Female**	15.30	0.61	0.44–0.85**	17.07	1.01	0.79–1.31	14.51	0.83	0.62–1.10	15.54	0.87	0.63–1.20
**Working Status**	X^2^ = 0.00			X^2^ = 4.29^Ψ^			X^2^ = 0.50			X^2^ = 18.81^ΨΨ^		
**Not Working**	1.00	Ref		2.58	Ref		3.71	Ref		6.70	Ref	
**Working**	99.00	1		97.42	0.54	0.29–1.02	96.29	1.46	0.91–2.34	93.30	0.42	0.24–0.75**
**Employment status**	X^2^ = 1.21			X^2^ = 0.55			X^2^ = 12.29^Ψ^			X^2^ = 26.41^ΨΨ^		
**Seasonal**	4.26	Ref		19.00	Ref		12.49	Ref		22.46	Ref	
**All year**	91.82	0.96	0.57–1.63	71.69	1.09	0.80–1.50	70.50	0.90	0.69–1.19	63.71	1.23	0.92–1.64
**Occasional**	3.93	0.73	0.34–1.58	9.30	1.17	0.75–1.82	17.01	1.04	0.72–1.50	13.82	1.58	1.03–2.45*
**Respondent earning gap**	X^2^ = 7.80			X^2^ = 20.85^ΨΨ^			X^2^ = 28.84^ΨΨΨ^			X^2^ = 0.27		
**Partner no income**	4.23	Ref		0.77	Ref		1.21	Ref		1.33	Ref	
**More than**	13.41	1.22	0.54–2.74	10.7	2.06	0.51–8.29	10.65	1.10	0.45–2.68	4.90	0.97	0.37–2.53
**Less than**	70.83	0.92	0.46–1.84	81.98	1.09	0.28–4.23	75.76	0.76	0.32–1.77	83.36	1.15	0.49–2.71
**Same**	11.52	0.96	0.46–2.00	6.54	1.00	0.25–3.99	12.38	0.54	0.23–1.26	10.41	1.09	0.45–2.66
**Respondent autonomy**	X^2^ = 12.24			X^2^ = 26.96^ΨΨ^			X^2^ = 115.62^ΨΨΨ^			X^2^ = 3.00		
**Respondent alone**	6.24	Ref		2.75	Ref		3.02	Ref		2.00	Ref	
**Respondent and partner**	38.54	0.52	0.27–1.00*	12.22	0.38	0.18–0.82*	41.24	1.09	0.58–2.05	37.48	0.61	0.26–1.44
**Partner alone**	53.17	0.55	0.29–1.06	84.09	0.70	0.35–1.41	55.47	2.23	1.22–4.07**	59.93	0.80	0.34–1.88
**Someone else**	2.04	0.88	0.18–4.34	0.95	0.81	0.24–2.74	0.27	1.51	0.34–6.74	0.58	1.03	0.18–5.75
**Spending decision**	X^2^ = 14.80^Ψ^			X^2^ = 0.58^Ψ^			X^2^ = 68.74^Ψ^			X^2^ = 3.34		
**Respondent alone**	43.4	Ref		74.34	Ref		27.78	Ref		54.36	Ref	
**Respondent and Partner**	37.41	0.78	0.54–1.11	17.42	1.13	0.84–1.51	60.10	0.65	0.50–0.86**	37.78	0.87	0.63–1.19
**Partner alone**	17.15	0.86	0.60–1.23	7.98	1.00	0.65–1.54	12.12	0.40	0.28–0.57***	7.74	0.90	0.56–1.43
**Someone else**	2.05	2.30	0.82–6.43	0.26	1.81	0.25–13.26	0.00	1.00		0.13	1.00	
**Total**	976.00			848.00			1301.00			976.00		

The prevalence of empowered women varies greatly across regions, as shown in (Table 3). Sub-Saharan Africa displays wide disparities, with higher levels in Zimbabwe (50.46%, 95% CI: 48.41%−52.51%) but much lower levels in Mali (2.95%, 95% CI: 2.15%−4.03%). In South and Southeast Asia, Cambodia has the highest prevalence at 52.94% (95% CI: 51.31%−54.55%), while Afghanistan shows the lowest at 10.39% (95% CI: 8.97%−12.01%). Central Asia shows strong empowerment levels in the Kyrgyz Republic (61.52%, 95% CI: 58.59%−64.36%), and Latin America and the Caribbean leads in empowering women as Honduras (64.23%, 95% CI: 62.76%−65.67%) showing the highest figures across all continental regions.

**Table 3:** Prevalence of Empowered Women by Continental Regions

Continental Regions	Country	Empowered N (%)	95% CI (%)
**Sub-Sahara Africa**	Angola 2015–16	1223 (49.20)	47.30–51.24
	Benin 2017–18	797 (30.98)	29.23–32.80
	Burundi 2016–17	660 (27.49)	25.74–29.32
	Cameroon 2018	878 (43.48)	41.33–45.65
	Comoros 2012	157 (24.94)	21.71–28.48
	Cote d’Ivoire 2021	470 (29.58)	27.39–31.88
	Ethiopia 2016	273 (32.50)	29.41–35.75
	Gambia 2019–20	164 (18.17)	15.79–20.83
	Liberia 2019–20	385 (49.22)	45.72–52.72
	Madagascar 2021	1449 (48.04)	46.26–49.83
	Malawi 2015–16	538 (48.72)	45.78–51.67
	Mali 2018	38 (2.95)	2.15–4.03
	Nigeria 2018	1855 (37.73)	36.38–39.09
	Sierra Leone 2019	287 (24.37)	21.99–26.90
	Tanzania 2015–16	462 (39.51)	36.74–42.34
	Togo 2013–14	709 (25.48)	23.89–27.13
	Uganda 2016	1130 (32.86)	31.30–34.44
	Zambia 2018	1005 (39.76)	37.87–41.69
	Zimbabwe 2015	1154 (50.46)	48.41–52.51
**South and Southeast Asia**	Afghanistan 2015	163 (10.39)	8.97–12.01
	Cambodia 2021–22	1926 (52.94)	51.31–54.55
	Myanmar 2015–16	614 (34.52)	32.35–36.76
	Nepal 2022	615 (44.58)	41.97–47.22
**Central Asia**	Kyrgyz Republic 2012	672 (61.52)	58.59–64.36
**Latin America and the Caribbean**	Haiti 2016–17	1512 (62.62)	60.67–64.53
	Honduras 2011–12	2674 (64.23)	62.76–65.67

### Women’s empowerment as a protective factor against IPV across countries

**Fig. 2:**
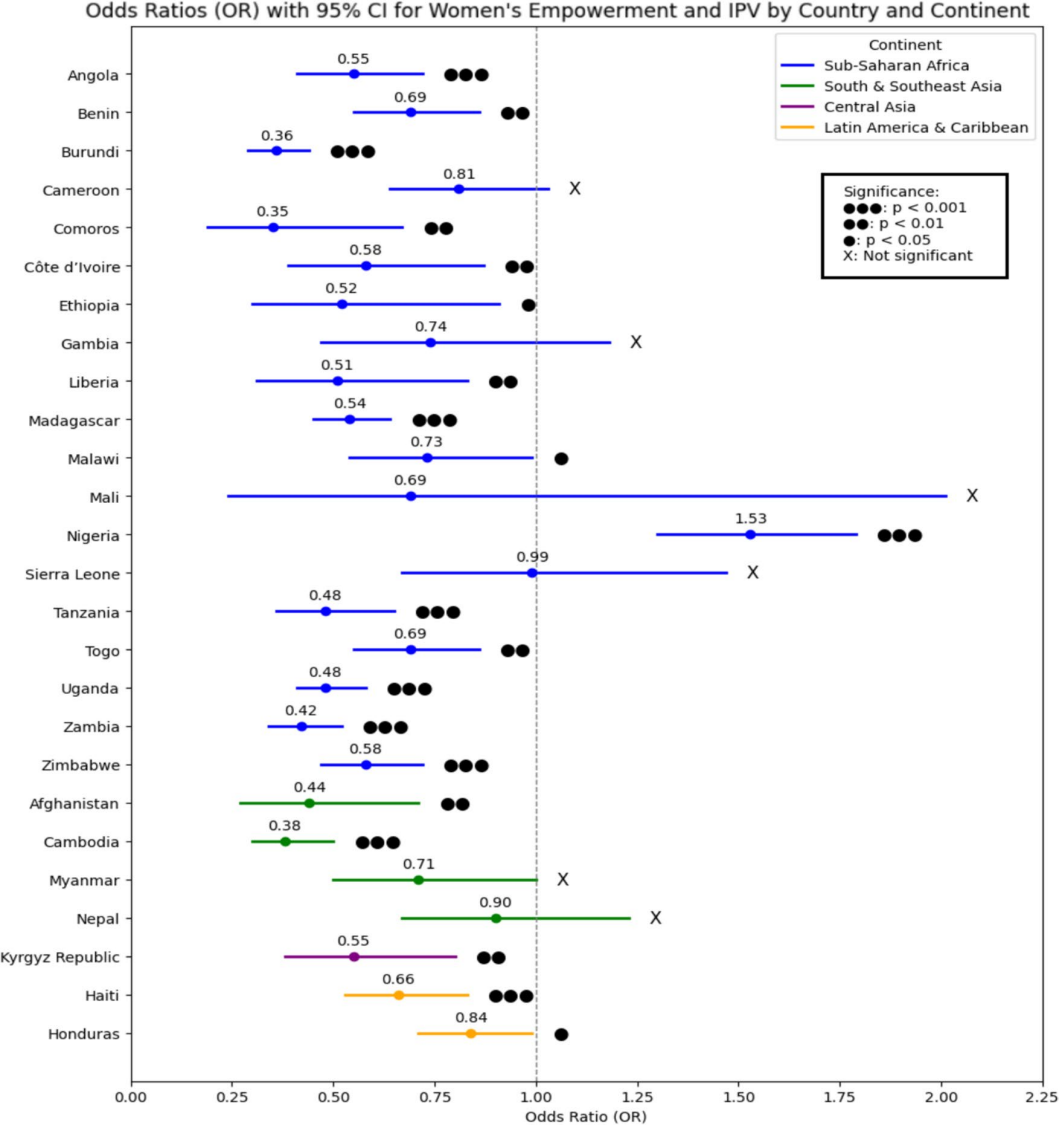
Forest plot showing odds ratios (ORs) and 95% confidence intervals (CI) for associations between women’s empowerment and intimate partner violence (IPV) across countries and continents

The relationship between women’s empowerment and IPV was analyzed using survey-weighted logistic regression, to explore the direct association between the two variables. Empowerment generally worked as a protective factor against IPV across most countries, except in Nigeria, where it was associated with increased risk (OR: 1.53, 95% CI: 1.30–1.79, *p* < 0.001). In Sub-Saharan Africa, significant protective effects were observed in Angola (OR: 0.55, 95% CI: 0.41–0.72, *p* < 0.001), Burundi (OR: 0.36, 95% CI: 0.29–0.44, *p* < 0.001), Tanzania (OR: 0.48, 95% CI: 0.36–0.65, *p* < 0.001), Uganda (OR: 0.48, 95% CI: 0.41–0.58, *p* < 0.001), and Zambia (OR: 0.42, 95% CI: 0.34–0.52, *p* < 0.001). Similar protective trends were observed in South and Southeast Asia, with Afghanistan (OR: 0.44, 95% CI: 0.27–0.71, *p* < 0.01) and Cambodia (OR: 0.38, 95% CI: 0.30–0.50, *p* < 0.001) showing statistically significant effects. In Central Asia, the Kyrgyz Republic (OR: 0.55, 95% CI: 0.38–0.80, *p* < 0.01) exhibited protective associations, while in Latin America and the Caribbean, Haiti (OR: 0.66, 95% CI: 0.53–0.83, *p* < 0.001) and Honduras (OR: 0.84, 95% CI: 0.71–0.99, *p* < 0.05) demonstrated reduced risks. As shown in (Fig. 2), these findings highlight the overall protective role of empowerment against IPV across regions, with Nigeria being the notable exception.

## Discussion

The high occurrence of IPV is deeply concerning and requires urgent attention. The prevalence of IPV varies significantly across countries, highlighting the intricate connections between socio-economic, and demographic factors.

In Sub-Saharan Africa, the prevalence of IPV against working women varies considerably. Sierra Leone shows the highest rate at 69.61%, while Comoros shows the lowest at 20.51%. This stark contrast reflects not only differences in prevalence but also the varying degrees of awareness, social norms, and legal frameworks across countries [56]. Liberia and Mali stand out at 66.54% and 53.39%, respectively. These figures underscore the urgency of addressing IPV as a public health and human rights concern in these nations. The high prevalence in these countries indicates deeply ingrained gender inequalities and the need for comprehensive interventions.

In South and Southeast Asia, Afghanistan shows the highest rate at 54.41%, highlighting women’s challenges in this conflict-affected region. Cambodia, with the lowest rate at 12.81%, represents a different context altogether. These disparities illustrate how domestic conflict, social norms, and economic opportunities impact IPV prevalence. Several factors, including access to education and economic opportunities, contribute to the variations observed. The Kyrgyz Republic in Central Asia has a rate of 30.92%, reflecting the challenges faced by countries in that region regarding violence and gender equality. These figures emphasize the need for strategies tailored to each region. The collective findings paint a concerning picture of violence against women, with an alarming 39.46% experiencing violence across all regions.

Looking beyond the prevalence, a closer examination of socio-demographic factors reveals complex patterns associated with IPV. Age plays a role in this matter; for instance, women aged 40–44 and 45–49 in Uganda face higher odds of experiencing IPV, highlighting the vulnerability of older women. Conversely, in Gambia, women aged 35 to 39 have odds of facing such violence, indicating potential protective factors within this age group. The residence type also offers intriguing insights. Urban areas often demonstrate higher odds of IPV in countries like Malawi, Tanzania, Haiti, and Honduras. This urban–rural divide underscores the importance of understanding the unique challenges that IPV can face by women in both settings [57].

The relationship between the wealth index and IPV varies across countries which are in some nations like Uganda, Zambia, and Myanmar, individuals in the richest wealth index category have significantly lower odds of experiencing IPV. However, Madagascar, Sierra Leone, and Honduras exhibit contrasting trends, with individuals in the poorest wealth index category displaying significantly lower odds of experiencing IPV. These disparities highlight the complex interplay between wealth and IPV. Education levels also play a multifaceted role in domestic violence. In some countries, such as Cameroon, having completed or attained secondary education is linked to increased chances of experiencing IPV. In contrast, countries like Cambodia, Nepal, Haiti, and Honduras show that individuals with primary and secondary education consistently display lower odds of experiencing IPV. The different examples highlight the importance of targeted interventions that consider how education can empower women and challenge expectations around gender [58].

Shifting our focus to how women’s empowerment relates to violence, we discovered that financial independence and being part of the workforce can act as protective factors. This finding highlights the critical role of economic empowerment in reducing vulnerability to violence. The employment type also plays a significant role in the risk of IPV. For instance, Madagascar and Zambia show that individuals with occasional employment exhibit significantly higher odds of experiencing IPV than those with seasonal employment. Stable year-round employment, as seen in Nigeria and Honduras, appears to have a protective effect. These nuanced associations emphasize the importance of employment stability and job security in empowering women to escape abusive situations [59]. Earning disparity between partners reveals intriguing patterns. In Tanzania and Afghanistan, women who earn less than their partners or the same amount as their partners experience lower odds of IPV. This suggests that financial parity within a relationship may reduce IPV risk. These findings challenge traditional gender roles and highlight the importance of economic equality within partnerships.

Respondent autonomy in decision-making presents nuanced associations with IPV. In Burundi and Cambodia, women whose partners make decisions alone face significantly higher odds of experiencing IPV, whereas women who make decisions alone serve as a reference. However, in Nigeria and Zambia, women whose partners assume decision-making autonomy are less likely to experience IPV. The relationship between spending decisions and IPV is complex, with collaborative spending decisions associated with both increased and decreased IPV risk depending on the country. Honduras presents a unique scenario where collaborative spending decisions are linked to lower odds of IPV. So, Addressing the issue of violence necessitates implementing approaches that question societal norms, encourage women’s economic empowerment, and safeguard their autonomy in decision-making.

The prevalence of women’s empowerment varies significantly across regions, reflecting global patterns. Higher levels in Latin America, such as in Honduras, may result from improved education and greater economic opportunities for women [60]. In contrast, the lower prevalence observed in Sub-Saharan Africa, particularly in Mali, suggests enduring cultural and structural barriers to women’s empowerment, consistent with findings from other studies [61].

The findings also indicate that women’s empowerment generally reduces IPV risk across regions, with strong protective effects in countries like Burundi, Tanzania, and Cambodia. However, Nigeria stands out as an exception, where empowerment is linked to a higher risk of IPV (OR: 1.53, 95% CI: 1.30–1.79, *p* < 0.001). This may be due to resistance against shifting gender norms in patriarchal societies, where male dominance is challenged. In Nigeria, cultural stigmatization of empowered women increases their vulnerability to IPV, as shifts in gender roles often provoke resistance. Such resistance can lead to a backlash, where men use violence to reassert control, a pattern observed in other highly patriarchal societies [62].

### Limitations

It is important to acknowledge that there remain some limitations in this study. One major limitation is that the data used for IPV and women’s empowerment variables rely on self-reported information from DHS surveys, which may introduce biases such as social desirability, dominance effects, or recall errors, potentially impacting data accuracy. The self-reported nature of the data may also result in underreporting of IPV due to stigma or fear of repercussions, particularly in regions where discussing such issues is culturally sensitive. Additionally, another limitation arises from the varying time periods of the dataset across countries spanning from 2011 to 2022, as historical, political, and socio-economic contexts may have shifted over time. For instance, changes such as legal reforms, policy interventions, or awareness campaigns addressing IPV and empowerment could influence reporting across countries. The cross-sectional nature of the data further limits the ability to establish causal relationships between women’s empowerment and IPV, as the findings represent associations rather than definitive causation. Furthermore, the way in which women’s empowerment is measured can vary between countries and cultures due to differences in social and religious norms. These variations can impact the reliability and comparability of the data, as the operationalization of empowerment in this study primarily focuses on economic and decision-making aspects, potentially overlooking other critical dimensions such as psychological or emotional empowerment. The unequal sample sizes for each country may also affect the result precision and representativeness. Countries with larger samples had more statistical power and less sampling error. Additionally, differences in the diversity of samples across countries can impact the accuracy of the results, which in turn limits the applicability of the findings to each country’s circumstances. Lastly, due to data insufficiency, we had to omit many countries from our analysis. Future studies should consider using standardized, reliable data sources that consistently measure IPV and women’s empowerment across countries and cultures. Larger, more representative samples that reflect each country’s population diversity and complexity should also be used for better research outcomes.

## Conclusion

This study provides evidence that empowering women socially through opportunities for employment and autonomy is associated with reducing the prevalence of IPV. The analysis highlights significant demographic factors influencing vulnerability to IPV, with older women, urban residents, and individuals living below the poverty line or lacking educational backgrounds being at higher risk. Empowerment indicators such as economic independence, employment status, equal earnings, and decision-making autonomy show protective associations against IPV. These findings underscore the potential role of economic and social empowerment in mitigating IPV while acknowledging that empowerment alone may not address all underlying causes. It is crucial for policymakers, organizations, and communities to utilize these findings to create more comprehensive environments for working women, fostering safety and equality in their lives.

## Data Availability

Availability of data and materials: Data is available from the Demographic and Health Surveys (DHS) program. https://dhsprogram.com.

## Abbreviations

AOR: Adjusted odds ratio
CI: Confidence interval
DHS: Demographic and health survey
EA: Enumeration area
IPV: Intimate partner violence
IR: Individual recorded
